# Stimulus Statistics Change Sounds from Near-Indiscriminable to Hyperdiscriminable

**DOI:** 10.1371/journal.pone.0161001

**Published:** 2016-08-10

**Authors:** Christian E. Stilp, Keith R. Kluender

**Affiliations:** 1 Department of Psychological and Brain Sciences, University of Louisville, Louisville, Kentucky, United States of America; 2 Department of Speech, Language, and Hearing Sciences, Purdue University, West Lafayette, Indiana, United States of America; McGill University Department of Physiology, CANADA

## Abstract

Objects and events in the sensory environment are generally predictable, making most of the energy impinging upon sensory transducers redundant. Given this fact, efficient sensory systems should detect, extract, and exploit predictability in order to optimize sensitivity to less predictable inputs that are, by definition, more informative. Not only are perceptual systems sensitive to changes in physical stimulus properties, but growing evidence reveals sensitivity both to relative predictability of stimuli and to co-occurrence of stimulus attributes within stimuli. Recent results revealed that auditory perception rapidly reorganizes to efficiently capture covariance among stimulus attributes. Acoustic properties *per se* were perceptually abandoned, and sounds were instead processed relative to patterns of co-occurrence. Here, we show that listeners’ ability to distinguish sounds from one another is driven primarily by the extent to which they are consistent or inconsistent with patterns of covariation among stimulus attributes and, to a lesser extent, whether they are heard frequently or infrequently. When sounds were heard frequently and deviated minimally from the prevailing pattern of covariance among attributes, they were poorly discriminated from one another. In stark contrast, when sounds were heard rarely and markedly violated the pattern of covariance, they became hyperdiscriminable with discrimination performance beyond apparent limits of the auditory system. Plausible cortical candidates underlying these dramatic changes in perceptual organization are discussed. These findings support efficient coding of stimulus statistical structure as a model for both perceptual and neural organization.

## Introduction

Objects and events in the sensory environment are highly predictable, making most of the energy impinging upon sensory transducers redundant. According to the Efficient Coding Hypothesis [1–2], the role of early sensory processing is to detect, extract, and exploit predictability in the input. An efficient sensory system should not only weaken its response to frequent or expected stimuli, but also produce a stronger response to infrequent or novel stimuli [3]. Seizing upon predictability in the environment optimizes sensitivity to unpredictability–informative change that facilitates adaptive behavior [4].

Animal and human studies alike reveal heightened sensitivity to infrequent (less predictable) stimuli. Single-unit physiological studies demonstrate increased neural firing in response to a low-probability change in the stimulus, known as stimulus-specific adaptation (SSA; inferior colliculus: [5–6]; thalamus: [7–8]; cortex: [9–11]). Similar (but not identical) mechanisms are reported at the neural population level in the event-related cortical potential termed the mismatch negativity response (MMN; [12–15]). In both cases, unpredictable (‘deviant’) inputs elicit higher firing rates or larger evoked responses than predictable (‘standard’) inputs. Sensitivity to stimulus novelty extends to behavior as well, as discrimination is superior for rarely presented sounds [16].

While widely studied, probability of occurrence is only one form of predictability in the environment (*e*.*g*., covariance among stimulus features, conditional and transitional probabilities across time). Additionally, while natural sounds are typically complex and vary along a multitude of physical dimensions, stimuli used in these foregoing studies were relatively simple sounds that varied along a single physical dimension. Most natural signals are comprised of multiple attributes that covary in ways that reflect a structured world. For example, many acoustic attributes of speech sounds covary with one another in ways that reflect constraints on vocal tracts, and this redundancy provides impressive perceptual resilience to signal distortion [17–22].

Covariance among stimulus properties has dramatic consequences for perceptual organization. For example, a lifetime of experience with robust covariance between binocular disparity and texture leads to these cues functioning as the single dimension of perceived slant [23]. Perceptual reorganization to efficiently capture covariance among attributes of novel sounds is sufficiently robust to develop within minutes of hearing them [24–26]. When presented with a range of novel complex sounds with near-perfectly redundant acoustic properties, discrimination performance was best predicted by whether stimulus differences adhered to or violated the main pattern of covariance among stimulus attributes (*i*.*e*., according to shared versus unshared covariance). As evidence of perceptual reorganization, sounds that are consistent with the main pattern of covariance remained discriminable, but sounds that modestly violated this pattern were poorly discriminated despite all stimuli being matched for equivalent psychoacoustic differences. Values for individual stimulus dimensions were not atypical; only their combinations varied in probability.

To the extent that enhancing transmission of information increases efficiency of sensorineural systems, heightened detection of changes from predictable occurrences of stimuli and from predictable co-occurrences of stimulus attributes are both expected. However, while large unidimensional physical deviations perceptually ‘pop out’, nothing is known about perception of large deviations from statistical context defined by covariance among attributes. Here, we investigate whether increasingly large deviations from experienced patterns of covariance receive privileged perceptual processing like that demonstrated for deviations (*i*.*e*., novelty) from predictable presentations of simple sounds. Magnitudes of novelty responses increase with increasing unidimensional dissimilarity between ‘standard’ and ‘deviant’ sounds [9,15]. Do complex sounds with properties that are increasingly *statistically* dissimilar become better discriminated?

**Fig 1:**
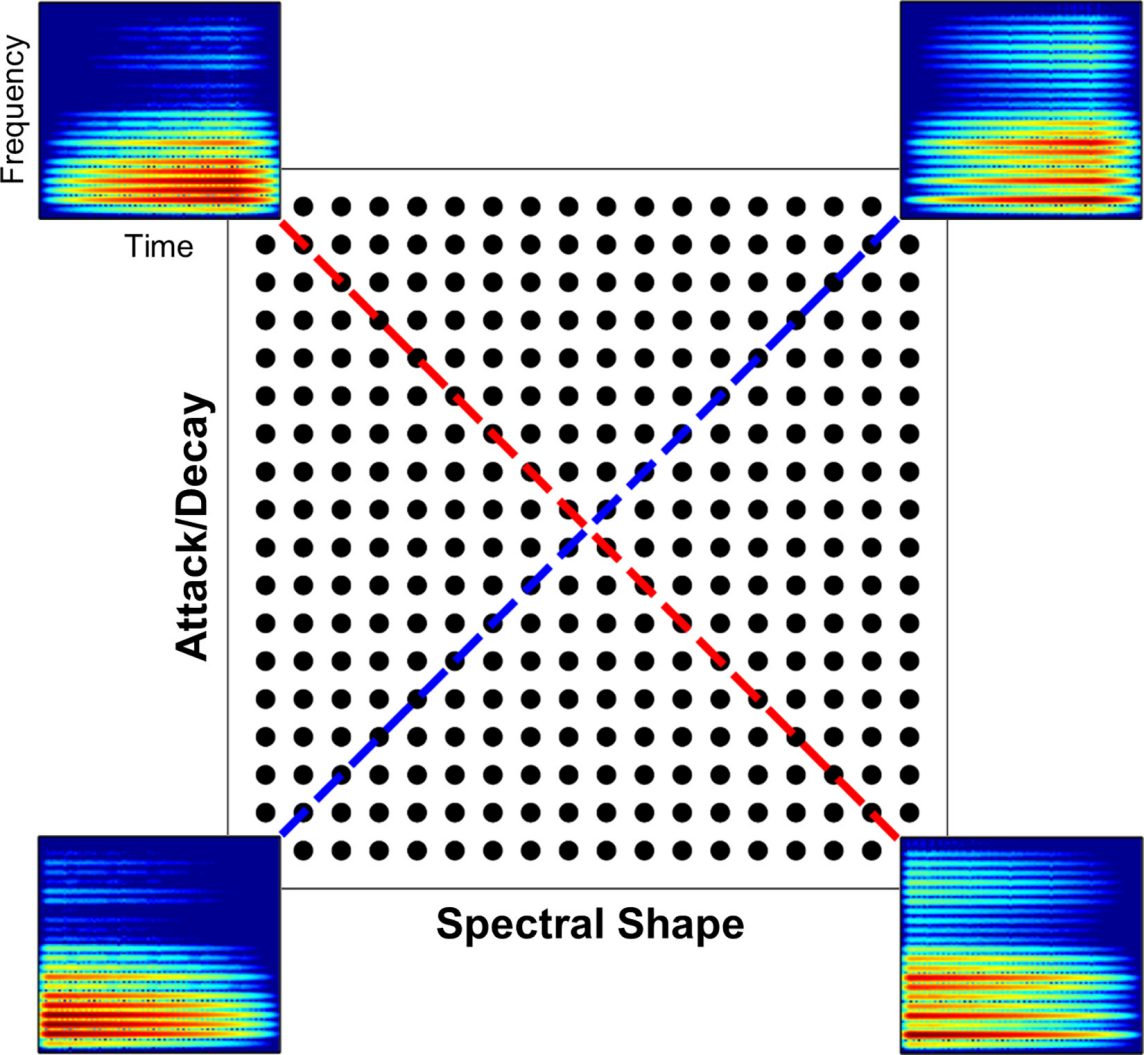
Stimulus matrix. Each circle represents one stimulus; different subsets from this matrix were presented in each experiment. Corner stimuli are replaced by spectrograms (500-ms abscissa, 10 kHz ordinate) to illustrate variation in Spectral Shape and Attack/Decay. Covariance between these properties occurs along either the Consistent statistical dimension (blue line) or the Orthogonal dimension (red line). Each experiment was counterbalanced such that half of listeners heard Consistent stimuli along the blue vector and Orthogonal stimuli along the red vector, while the other half heard Consistent stimuli along the red vector and Orthogonal stimuli along the blue vector.

The present experiments employed novel complex sounds (Fig 1) to explore perceptual organization based upon both lower-order (probability of occurrence) and higher-order statistical properties (covariance among stimulus attributes). We hypothesized that by making stimuli increasingly unpredictable, both by decreased probability of occurrence and larger violations of covariance among acoustic attributes, they would become more discriminable. Discriminability improved with larger violations of the principal pattern of covariance among attributes, demonstrating a close relation between perceptual organization and experienced statistics of the sensory environment. When sounds were infrequent and were extreme violations of predictable patterns of covariance, they became hyperdiscriminable with perceptual performance beyond apparent limits of the auditory system (*i*.*e*., discrimination performance based on acoustic differences alone).

## Results

### Behavioral Results

**Fig 2:**
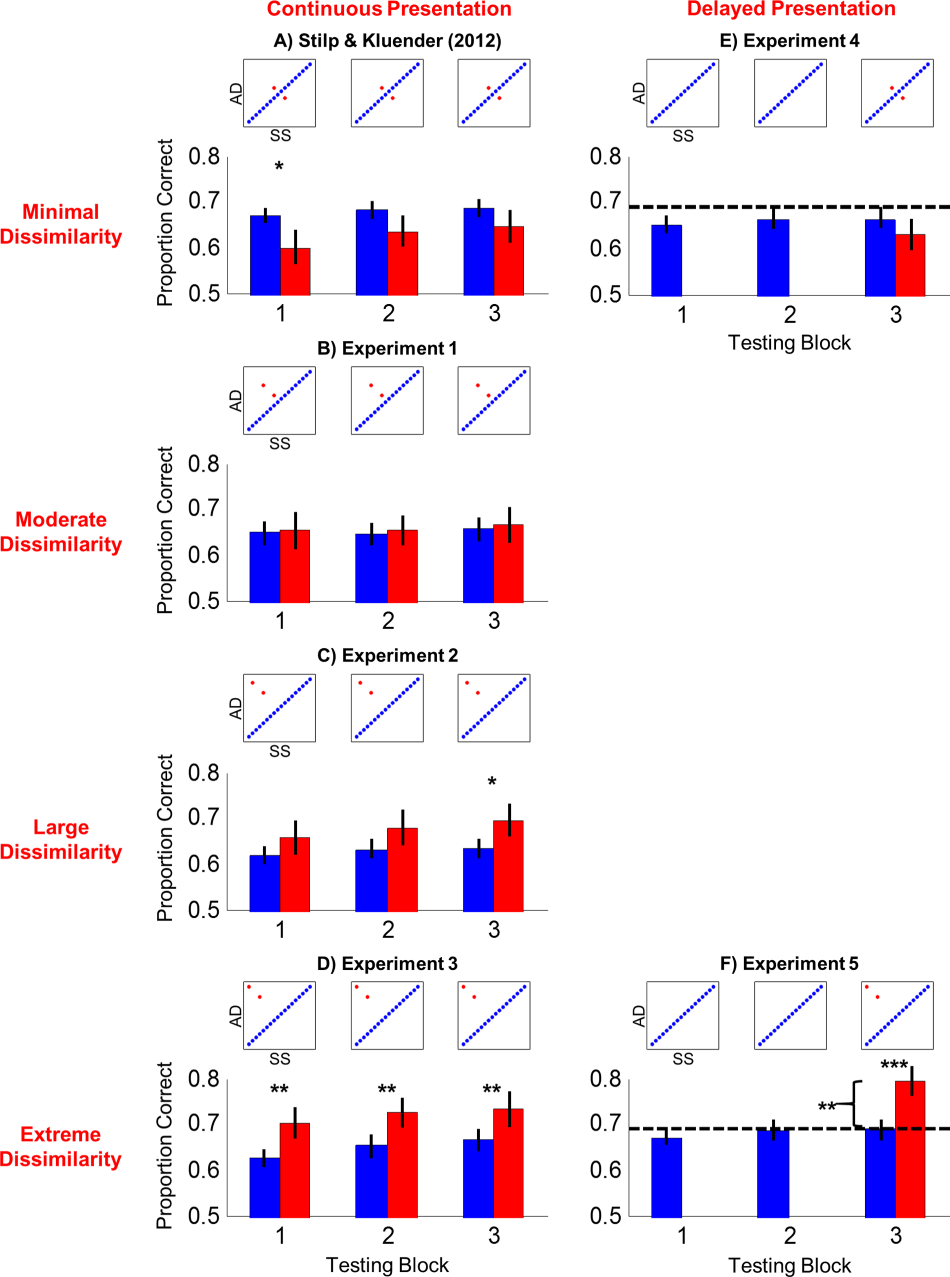
Stimulus discriminability is modulated by statistical structure among acoustic properties. Figures plot mean accuracy for discriminating pairs of Consistent (blue) or Orthogonal sounds (red) as a function of testing block for each experiment. Insets depict stimulus matrices to indicate which stimuli were tested in each block of each experiment. Half of the participants in each experiment heard stimuli as depicted while the other half heard counterbalanced stimuli rotated 90°. Rows are arranged according to statistical properties of Orthogonal sounds (red text) indicating the extent to which they violated the prevailing pattern of covariance supported by the Consistent sounds, increasing progressively from Minimal Dissimilarity (top row; inferior discrimination) to Extreme Dissimilarity (bottom row; superior discrimination). Major columns indicate frequency of presentation for Consistent and Orthogonal sound pairs: equally often (left column) or Orthogonal sounds withheld until the third testing block (right column). Dashed lines represent baseline performance when acoustic dimensions shared zero redundancy (mean accuracy = 0.690 [24]); significant improvement beyond baseline performance in Experiment 5 indicates hyperdiscriminability. Asterisks indicate statistically significant differences; *P < .05, **P < .01, ***P < .001. Error bars indicate standard error of the mean.

The first question at test is how perception organizes in response to deviations of increasing magnitude from the principal pattern of covariance among stimulus attributes. When deviations were very small (*i*.*e*., minimal violations of the pattern of covariance supported by Consistent sounds, [26]), listeners were nearly unable to discriminate Orthogonal sounds with performance falling to near-chance levels (mean proportion of pairs correctly discriminated = 0.600, standard error of the mean [s.e.] = .033; compared to mean accuracy for Consistent pairs = 0.670, s.e. = .014; *Z* = 2.527, *P* = .011; Fig 2A). This difference extinguished with further testing (Block 2: Consistent mean = 0.681, s.e. = .016, Orthogonal mean = 0.634, s.e. = .030; Block 3: Consistent mean = 0.687, s.e. = .016, Orthogonal mean = 0.647, s.e. = .033). Here, we manipulated shared and unshared covariance by positioning Orthogonal sound pairs at increasing distances away from Consistent stimuli on the diagonal of the stimulus matrix. This systematically increased the amount of unshared covariance in the stimuli, making pairs increasingly statistically deviant.

As the magnitudes of statistical deviations increased, discrimination of those sounds improved from being comparable (Experiment 1 Block 1: Consistent mean = 0.649, s.e. = .021, Orthogonal mean = 0.653, s.e. = .036; Block 2: Consistent mean = 0.648, s.e. = .021, Orthogonal mean = 0.653, s.e. = .027; Block 3: Consistent mean = 0.656, s.e. = .022, Orthogonal mean = 0.666, s.e. = .036; Fig 2B) to better than that for Consistent sounds (Experiment 2 Block 1: Consistent mean = 0.617, s.e. = .017, Orthogonal mean = 0.656, s.e. = .035; Block 2: Consistent mean = 0.631, s.e. = .018, Orthogonal mean = 0.678, s.e. = .038; Block 3: Consistent mean = 0.632, s.e. = .019, Orthogonal mean = 0.694, s.e. = .032; *Z* = 2.292, *P* = .022; Fig 2C; S1 Table). Superior discrimination of maximally statistically deviant Orthogonal sounds persisted throughout Experiment 3 (Block 1: Consistent mean = 0.628, s.e. = .016, Orthogonal mean = 0.703, s.e. = .029 [*Z* = 2.945, *P* = .003]; Block 2: Consistent mean = 0.652, s.e. = .021, Orthogonal mean = 0.725, s.e. = .029 [*Z* = 2.972, *P* = .003]; Block 3: Consistent mean = 0.665, s.e. = .021, Orthogonal mean = 0.734, s.e. = .035 [*Z* = 2.622, *P* = .009]; Fig 2D).

The second question at test is whether enhanced processing of unexpected (infrequent) occurrences extends beyond single acoustic dimensions to derived perceptual dimensions capturing patterns of covariance between stimulus attributes. Two experiments introduced manipulation of surprisal [27–28] by withholding presentation of Orthogonal sound pairs until the third and final testing block. These unexpected Orthogonal sound pairs deviated from the main pattern of covariance by either minimal (Experiment 4) or maximal amounts (Experiment 5). When sounds were unexpected but minimally deviant in terms of covariance, they were discriminated modestly worse than Consistent sound pairs (Consistent mean = 0.663, s.e. = .017, Orthogonal mean = 0.628, s.e. = .030; related-samples Wilcoxon signed-rank test: *Z* = 1.371, *P* = .170; Fig 2E), similar to when these sounds were presented as frequently as other sounds throughout the experiment (Fig 2A).

Conversely, highly statistically deviant sounds that were both unexpected and extreme violations of feature covariance were discriminated extremely well (mean = 0.795, s.e. = .028). Performance was significantly better than: Consistent sounds (mean = 0.690, s.e. = .018; related-samples Wilcoxon signed-rank test: *Z* = 3.650, *P* = .0003; Fig 2F); the same Orthogonal sounds with exposure equal to that for other sounds (Experiment 3 Block 1: mean = 0.703, s.e. = .028; one-tailed Mann-Whitney U test: *U* = 2.200, *P* = .014; Fig 2D); and most importantly, baseline performance when stimuli do not share redundant attributes (one-sample one-tailed Wilcoxon signed-rank test against mean discrimination accuracy of 0.690: *Z* = 2.590, *P* = .005; dashed line in Fig 2F). Highly statistically deviant sounds were hyperdiscriminable with performance beyond apparent limits of auditory perception. Deferred presentation alone did not contribute to the hyperdiscriminability observed in Experiment 5, as Orthogonal trials in the final testing block of Experiment 4 were discriminated less accurately than Orthogonal trials in the final block of Experiment 5 (two-tailed Mann-Whitney U test: *U* = 3.724, *P* = .001). Relative predictability, by simple probability of occurrence and probability of co-occurrence between stimulus attributes, has dramatic consequences for perceptual organization, rendering sounds from near-indiscriminable to hyperdiscriminable.

### Computational Predictions

Principal components analysis (PCA) has reliably predicted discriminability on the basis of patterns of covariance between stimulus attributes [24, 26]. This same approach was used to predict behavioral performance in the present experiments. Values of Spectral Shape (SS) and Attack/Decay (AD) were coded as ordered pairs from 1 to 18 to indicate their positions along each axis of the stimulus matrix. These ordered pairs were arranged into matrices to represent the stimuli presented in each experiment. For example, stimuli in Experiment 1 were coded as follows: (1,1) to (18,18) for the Consistent stimuli, and (5,14) and (8,11) for the Orthogonal stimuli (see Fig 2B). This coding was repeated three times to represent stimuli being tested in three consecutive experimental blocks. A covariance matrix was computed on this stimulus list using the *cov* command in MATLAB (see Table 1 for covariance matrices for Experiments 1–5). Eigenvalues from PCA were calculated on these covariance matrices using the *eig* command in MATLAB (S2 Table). Experiment 1 from [26] (Fig 2A) served as a reference point, with substantial covariance along the Consistent dimension (λ_1_ = 49.27) and minimal covariance along the Orthogonal dimension (λ_2_ = 0.46). Increasingly eccentric Orthogonal stimuli in Experiments 1–3 progressively increased the second Eigenvalue (Experiment 1: λ_2_ = 2.11, Experiment 2: λ_2_ = 7.05, Experiment 3: λ_2_ = 9.43), but presentation of the same Consistent stimuli resulted in an unchanged first Eigenvalue. Experiments 4 and 5 required a slightly modified approach as stimuli were no longer tested equally often. Therefore, ordered pairs representing the 18 Consistent stimuli were repeated three times (again to represent testing in all three experimental blocks) while ordered pairs representing the two Orthogonal stimuli were included only once (to represent testing in the third block alone). This marginally increased the first Eigenvalue (λ_1_ = 52.85) and decreased the second Eigenvalue relative to repeated presentations of the same stimuli (Experiment 4: λ_2_ = 0.16, compared to λ_2_ = 0.46 in [26]; Experiment 5: λ_2_ = 3.60, compared to λ_2_ = 9.43 in Experiment 3).

**Table 1:** Covariance matrices for experimental stimuli.

	1	2
Expt. 1	25.69	23.58
	23.58	25.69
Expt. 2	28.16	21.19
	21.19	28.16
Expt. 3	29.35	19.92
	19.92	29.35
Expt. 4	26.51	26.35
	26.35	26.51
Expt. 5	28.23	24.63
	24.63	28.23

Column headers indicate the first and second columns of the 2x2 covariance matrices calculated on stimuli presented in each experiment.

Previous experiments tested discriminability of Orthogonal sounds that deviated only modestly from the Consistent sounds, reflected by very small second Eigenvalues (length of second Eigenvector) of the covariance matrix. With relatively short Eigenvectors, larger second Eigenvalues corresponded to a decrease in the advantage in discriminability for Consistent versus Orthogonal sound pairs. Across wide differences in stimulus selection [26], as relatively more covariance lay along the Orthogonal dimension, Orthogonal sound pairs were discriminated increasingly well relative to Consistent pairs, approaching parity.

The same PCA model predicts that, beyond the range tested, discriminability of Orthogonal stimuli should improve as the length of the second Eigenvector is further increased (larger Eigenvalue). For larger second Eigenvalues, PCA predicts that discriminability of Orthogonal pairs should exceed that for Consistent pairs even approaching hyperdiscriminability, and that prediction is tested here.

**Fig 3:**
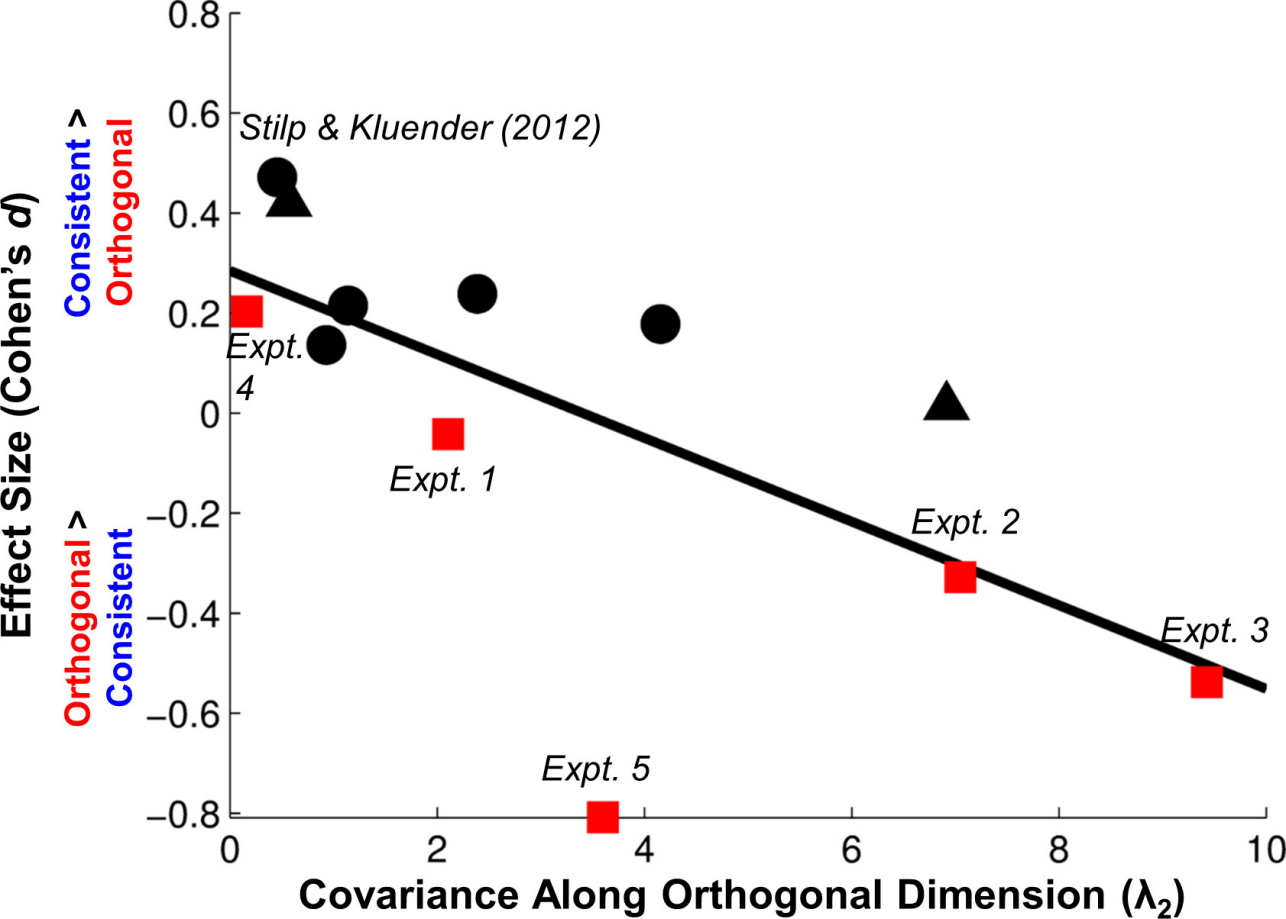
Using stimulus statistics to predict behavioral discrimination. Covariance along the Orthogonal dimension in each experiment (as measured by the second Eigenvalue of the covariance matrix of tested stimuli, λ_2_) is along the abscissa, and effect size (Cohen’s *d*, calculated as the difference in mean discriminability between Consistent and Orthogonal stimuli, each averaged across experimental blocks) is along the ordinate. Positive values along the ordinate indicate Consistent stimuli were better discriminated than Orthogonal stimuli, while negative values indicate Orthogonal stimuli were better discriminated. Results from the present report are plotted as squares with each experiment labeled individually. Results from [24] are plotted as triangles, and results from [26] are plotted as circles. Experiment 1 from [26], which is included in Fig 2A as a point of reference, is the upper-leftmost circle, which is also labeled. The solid line is the linear regression fit. Increasing covariance along the Orthogonal dimension clearly results in those stimuli being better-discriminated, but results from Experiment 5 are an outlier such that rare, extreme deviations from stimulus statistics are discriminated far better than predicted by covariance alone.

The relationship between stimulus statistics and behavioral performance was assessed via linear regression (S3 Table). The second Eigenvalue of the covariance matrix of experimental stimuli (λ_2_) served as the predictor variable, and effect size (Cohen’s *d*, comparing mean discriminability of Consistent versus Orthogonal sound pairs; averaged across testing blocks) was the outcome variable. Fig 3 shows the regression across the present experiments (squares) as well as related experiments using the same stimuli ([24], triangles; [26], circles). Across all experiments in which all stimuli were tested equally often ([24,26], Experiments 1–3 here), stimulus statistics were highly correlated with behavioral performance (*R* = –0.871, *P* = .001).

Discrimination of Orthogonal pairs was relatively poor when acoustic attributes shared relatively little covariance (smaller λ_2_, positive effect sizes indicating Consistent stimuli were discriminated more accurately), but discriminability improved as Orthogonal stimuli conveyed greater covariance (larger λ_2_, negative effect sizes indicating Orthogonal stimuli were discriminated more accurately).

A second regression was conducted across all experiments, irrespective of whether all stimuli were tested equally often in an experiment or not. Inclusion of Experiments 4 and 5 in the regression markedly decreased correlation strength (*R* = –0.663, *P* = .019). While the regression is still statistically significant with performance in Experiment 4 adhering to the trend, results from Experiment 5 are a distinct outlier. The prediction error (squared residual) for this result is more than six times larger than any other prediction error in the analysis. While PCA predictions are consistent with trends across equivalent presentation formats, hyperdiscriminability discovered with late-appearing stimuli cannot be predicted by covariance alone, and instead requires inclusion of other stimulus statistics (frequency of occurrence; *i*.*e*., rarity).

## Discussion

Perception warped to capture stimulus statistical structure to an extreme not previously observed. Violating covariance between acoustic dimensions in complex sounds had profound effects on stimulus discriminability, ultimately resulting in hyperdiscriminability when presentations were deferred until the last block of presentations. Only one prior study reported very modest effects of violating a learned relationship between simple acoustic dimensions (frequency, intensity) in tone stimuli [29]. Simpson and colleagues [16] reported improved discrimination of noise bursts with rarely presented amplitudes or interstimulus intervals, and this improvement required sufficient acoustic dissimilarity to frequent sounds. Unlike previous work, individual values of physical dimensions AD and SS in the present study were never exceptional, as stimuli were distinct only with respect to co-occurrences of values of AD and SS. Discrimination of extremely deviant Orthogonal sounds improved when they were rare (Experiment 5), but this improvement only occurred when they were also sufficiently statistically dissimilar to frequently heard Consistent sounds (Experiment 4).

Neural novelty response magnitudes increase with increasing acoustic dissimilarity between ‘standards’ and ‘deviants’ [9,15]. Here, ‘deviant’ Orthogonal sounds were better discriminated with increasing statistical dissimilarity relative to the main pattern of covariance (‘standard’ Consistent sounds). Experimental methods vary widely across physiological, electrophysiological, and behavioral studies, but all results highlight general principles of novelty detection in response to changes from physical contexts and particularly statistical contexts in the present studies.

Past [24–26] and present results are consistent with the principle of non-isomorphism [30] whereby neural representations of sensory input along ascending neural pathways decreasingly resemble the input and better correspond to functionally significant stimulus properties. Neural coding becomes more statistically independent [31] and better captures emergent properties at higher levels [32]. Examples of non-isomorphic representations in auditory cortex include encoding spectral shape across varying absolute frequencies [33], relative changes in faster versus slower click trains [34–35], and relationships across frequency components instead of individual components [36–37]. Here, perceptual performance is predicted by statistical relationships between stimulus attributes while physical acoustic dimensions appear to be abandoned. While non-isomorphic transformations do not exclude parallel representations that more closely resemble physical stimulus properties (isomorphism [32]), present results reveal that relationships between acoustic dimensions are primary determinants of perceptual performance–not the acoustic dimensions themselves.

The present findings have special relevance for speech perception. Speech sounds are famously rich with statistical structure [38], and extracting stable relationships from highly variable inputs is critical to high-level perceptual processing including speech perception [13]. Multiple acoustic dimensions covary in adherence with lawful constraints upon vocal tracts [21,38]. For example, vowel sounds are well-characterized by peaks in the frequency spectrum (formants) which correspond to resonances in the vocal tract. As vocal tract length decreases systematically across adult men, adult women, and child talkers, laws of physical acoustics compel formant frequencies to increase proportionately. This relationship captures over 75% of variability in vowel productions across men, women, and children [21]. Reliable covariance between stimulus attributes has been proposed to underlie categorization in general [39] and contribute to categorical perception of complex sounds including speech [18,40].

Many have argued that probability of presentation is fundamental to perception and to categorization of complex sounds such as speech [40–43], even suggesting that, at best, other statistical regularities play secondary roles [44]. Here, performance was far better (but not exclusively) explained by covariance among stimulus properties. Discrimination of Orthogonal sounds improved as their statistical dissimilarity increased when probability of presentation was held constant (Experiments 1–3). Conversely, discriminability of minimally deviant Orthogonal sounds was similar when they were tested one-third (Experiment 4), one, three, or ten times as often as each Consistent sound pair [26]. Finally, discriminability of maximally deviant Orthogonal sounds was enhanced when they were tested less frequently (Experiment 5). Results require integration of probability of occurrence and patterns of covariance for perception, but with far greater importance attributed to covariance.

Stilp and colleagues [24] tested three simple connectionist models of neural organization to better understand effects of covariance among stimulus attributes when digression from the principal covariance was modest. A Hebbian [45] neural network model captured early aspects of listener performance, but predictably failed to adjust over time due to lack of inhibitory connections. An anti-Hebbian model [46] failed because it predicted enhanced discrimination of all violations of covariance, even modest violations for which decreased discriminability was observed. Closed-form PCA successfully predicted results from a wide range of experiments including Experiments 1–4 here. However, neither the closed-form nor connectionist implementation of PCA predicted the hyperdiscriminability observed in Experiment 5. This effect required that stimuli be unexpected due to lack of prior occurrence. As in everyday perception, perceptual organization reflects contributions of multiple concurrent statistical properties, and cannot be fully described by a single property.

Escera and Malmierca [47] proposed that the auditory system is hierarchically organized for novelty detection, with more complex levels of regularity encoded at higher levels of processing. Similarly, Kluender and Alexander [19] argued that processing of complex sounds is a progression of increasingly sophisticated processes for extracting predictable patterns, with hierarchical processing being a necessary consequence of successive relatively independent (efficient) representations. The neural locus or loci responsible for the present results remains an open question, but some neural observations are suggestive. Previous successes of a connectionist implementation of PCA [48] to predict results depend on inhibitory circuits from the output layer to input layers. In the microcircuitry across layers within cortical columns, such inhibitory signals may be provided in a fashion similar to that proposed to support predictive coding [49]. Less locally, required inhibitory circuitry may be provided within hierarchical auditory cortical regions, which extend from primary auditory cortex (AI) to belt areas to more lateral parabelt regions in a third stage of cortical processing [50]. While AI is responsive to most sounds, responses later in the auditory hierarchy are selective for more complex stimuli, such as band-limited noise and frequency-modulated sweeps in belt areas [51–53] and species-specific vocalizations such as human speech in parabelt areas [54].

Three important characteristics of cortical novelty responses make cortex an attractive neural locus for the observed behavioral results. First, acoustic similarity plays a larger role in cortical SSA than does simple probability. High acoustic similarity between standard and deviant stimuli extinguishes SSA despite extreme differences in probability of presentation (9:1 standard:deviant ratio; [9]). Here, statistical similarity (as defined by patterns of covariance) influenced stimulus discriminability far more than probability of presentation. Second, SSA in primary auditory cortex has been reported for complex sounds such as frozen noise and speech [11], offering some potential for SSA extending to more complex stimuli that are defined by predictable statistical structure. Third, the amplitude of the MMN response (generated in auditory cortex) increases with more repetitions of the standard stimulus before presenting the deviant [55]. Discriminability of maximally deviant Orthogonal sounds in Experiment 5 was enhanced following two blocks of Consistent-only testing, resulting in superior performance compared to the beginning of Experiment 4 when presentation of these sounds was not delayed. These promising parallels raise the possibility of “statistic-specific adaptation”, where stimulus discriminability is modulated by statistical relations among acoustic properties and not the properties (or specific stimuli) themselves. However, physiological investigations are needed in order to substantiate generalization from behavioral data.

Contemporary investigations of efficient coding [1–2] explore the statistics of natural stimuli and ways through which sensory systems capture this structure [56–58]. Here, principles of efficient coding captured dramatic changes in perceptual organization that reflected statistical properties of acoustic inputs, ultimately resulting in hyperdiscriminability. Results suggest efficient coding to be an underlying principle for both neural and perceptual organization.

## Materials and Methods

### Ethics Statement

All listeners provided written informed consent under protocols approved by the Institutional Review Board of the University of Wisconsin.

### Participants

One hundred ninety-nine undergraduate students participated in exchange for course credit (40 each in Experiments 1–4, 39 in Expt. 5). All self-reported normal hearing, and no one participated in more than one experiment.

### Stimuli

One waveform period (3.78 ms duration = 264 Hz fundamental frequency) was excised from recordings of a French horn and a tenor saxophone in the McGill University Music Database [59]. Pitch periods were iterated to 500-ms duration and matched in RMS energy. Attack/Decay (AD) was defined as the linear amplitude increase from zero at onset to peak amplitude (attack) before linear decrease to zero at offset (decay) without steady state. Attack durations were varied in eight 10-ms steps from 20 to 100 ms, and from 100 to 390 ms in nine equal logarithmic steps. Decays were 500 ms (total duration) minus attack duration. Spectral Shape (SS), defined as relative levels of energy across frequencies, varied via 18 summations of the two instrument endpoints in different proportions, ranging from 0.2 to 0.8 and summing to 1 across instruments. Mixture proportions were derived according to Euclidean distances between equivalent-rectangular-bandwidth-scaled [60] spectra processed by simulated auditory filters [61]. All stimulus processing was conducted in MATLAB. Human speech and musical instruments naturally vary in AD and SS, which are relatively independent both perceptually and in early neural encoding [62].

AD and SS were each exhaustively normed in two-alternative forced-choice (AXB) discrimination tasks until every pair of sounds separated by three stimulus steps was approximately equally discriminable for normal-hearing listeners. Dimensions were then fully crossed to create the stimulus matrix. A separate control study measured the discriminability of all stimulus pairs (separated by three stimulus steps along both AD and SS) along each main diagonal (red and blue lines in Fig 1). The result of this AXB discrimination control task was approximately equal discriminability across every pair of stimuli separated by a fixed distance (mean proportion correct = 0.690; [24]), thereby creating a perceptually linearized space. Experimental stimuli lay along either one main diagonal of the stimulus matrix, conforming to robust covariance between AD and SS (Consistent condition), or the perpendicular main diagonal (Orthogonal condition; see Fig 1).

### Experimental Design

Listeners discriminated sounds that were either *Consistent* with the main pattern of covariance between AD and SS or *Orthogonal* to this covariance. In each experiment, the vast majority of stimuli belonged to the Consistent condition (18 sounds, or 15 unique pairs of sounds) while a small number of stimuli formed the Orthogonal condition (two sounds, or one sound pair). In each case, sound pairs were separated by three stimulus steps along both AD and SS dimensions. Each trial presented one sound pair (either Consistent or Orthogonal) in a two-alternative forced-choice AXB triad with 250-ms ISIs. No feedback was provided regarding accuracy or whether Consistent or Orthogonal sounds were being presented. Within an experiment, each testing block consisted of either 128 trials (8 repetitions of each of the 15 Consistent sound pairs plus 8 repetitions of the one Orthogonal sound pair; Experiments 1–3 and final testing block of Experiments 4–5) or 120 trials (8 repetitions of the Consistent sound pairs only; first and second blocks in Experiments 4–5). Trials were tested in different random orders for each participant in each block.

Different subsets of this matrix were selected to define different degrees of shared versus unshared covariance between AD and SS. This was achieved by holding the Consistent dimension constant and selecting different pairs of Orthogonal sounds. In Stilp and Kluender [25] and Experiment 4, Orthogonal sounds were highly similar to the Consistent sounds by virtue of being positioned very close in the stimulus matrix (ordered pairs in Fig 2A and 2E: [8,11] and [11,8]). In Experiment 1, Orthogonal sounds were positioned slightly further away from Consistent stimuli (ordered pairs in Fig 2B: [5,14] and [8,11]). In Experiment 2, Orthogonal stimuli were positioned even further away (ordered pairs in Fig 2C: [2,17] and [5,14]). In Experiments 3 and 5, Orthogonal stimuli were positioned at the furthest distance possible from the Consistent stimuli in the stimulus matrix (ordered pairs in Fig 2D and 2F: [1,18] and [4,15]). Experiments were counterbalanced so half of listeners heard stimuli forming a positive correlation between AD and SS (as in Figs 1 and 2) while the other half heard stimuli forming a negative correlation (90° rotation of Figs 1 and 2). One group’s Orthogonal dimension was the other group’s Consistent dimension and *vice versa*, thus serving as its control and replication.

### Testing

Listeners participated in single-subject soundproof booths. Stimuli were upsampled to 48,828 Hz, D/A converted (Tucker-Davis Technologies RP2), amplified (TDT HB4), and played diotically at 72 dB SPL over circumaural headphones (Beyer-Dynamic DT-150). Participants heard trials in different randomized orders and responded by pushing labeled buttons on response boxes. Stimulus pairs were tested eight times in each of three testing blocks. Experiments 1–3 consisted of 384 trials (3 blocks of 128), lasting approximately 30 minutes. Experiments 4–5 consisted of 368 trials (first two blocks had 120 trials [Consistent pairs only], third block had 128 trials [Consistent and Orthogonal pairs]), lasting approximately 28 minutes. Participants were provided brief breaks between blocks.

### Statistical Analyses

Listeners discriminated pairs of sounds that were either Consistent with or Orthogonal to the dominant pattern of covariance among acoustic attributes. Omnibus analyses (ANOVA, Friedman test) are likely to result in Type II error when Orthogonal discriminability returns to (Experiment 1 in [26], Fig 2A) or begins at (Experiment 1 here, Fig 2B) a level matching Consistent discriminability. Instead, planned contrasts were employed to retain sensitivity to differences within a given experimental block. The difference between Consistent and Orthogonal discrimination within a given block was required to exceed a threshold of 5% before conducting statistical analyses, because this threshold reliably indicates significant differences between conditions in a given block [24,26].

Shapiro-Wilk tests were conducted to assess the normality of distributions of mean discrimination scores for Consistent and Orthogonal conditions. Distributions of mean Orthogonal scores were not normal (*i*.*e*., produced statistically significant Shapiro-Wilk tests), indicating that nonparametric analyses were appropriate. Nonparametric tests were conducted on paired samples (two-tailed Wilcoxon signed-rank test [*W*] comparing Consistent and Orthogonal performance in an experiment), independent samples (one- or two-tailed Mann-Whitney U tests [*U*] comparing Orthogonal performance across experiments and thus across listener groups), or one sample (one-tailed Wilcoxon signed-rank test [*W*] comparing discriminability against baseline performance when acoustic dimensions share zero redundancy, where mean proportion of trials correct = 0.690; [24]). Corrections for multiple comparisons on a single data set were made using Holm’s [63] method.

## Supporting Information

S1 Table Behavioral Results. Mean discrimination accuracy for every listener in each experiment depicted in Fig 2. Within a given experiment, each row depicts performance for a given listener. Means are calculated for Consistent and Orthogonal trials in each testing block. Group means and standard errors of the mean (SE) appear at the top of each section.
(XLSX)

S2 Table Covariance matrices and Eigenvalues for experimental stimuli. The leftmost column indicates the testing block (out of 3) in the experiment. For each experiment depicted in Fig 2, each stimulus is represented by the ordered pair indicating its position in the stimulus matrix, from (1,1) to (18,18). Within each experiment, the first column indicates position along the abscissa (Spectral Shape, SS) and the second column indicates position along the ordinate (Attack/Decay, AD). Within each testing block, Consistent stimuli are listed first and Orthogonal stimuli (when included) are listed second. Below these stimulus representations, the covariance matrix calculated on these stimuli is listed, followed by Eigenvalues of that covariance matrix. λ_1_ indicates the first Eigenvalue (corresponding to the Consistent dimension), and λ_2_ indicates the second Eigenvalue (corresponding to the Orthogonal dimension).
(XLSX)

S3 Table Predicting relative discriminability as a function of stimulus covariance. For 12 experiments including those in the present report, stimulus Eigenvalue, block means, overall means, and overall standard deviations are provided for Consistent and Orthogonal conditions. The second-to-last column lists pooled standard deviations across Consistent and Orthogonal conditions. The final column calculates Cohen’s effect size (d) for the difference in discriminating Consistent and Orthogonal stimuli (calculated as Consistent minus Orthogonal). Positive values indicate better performance when calculating Consistent stimuli, and negative values indicate better performance when discriminating Orthogonal stimuli. The bottom displays correlation coefficients between the λ_2_ and effect size for the first 10 experiments listed (where Consistent and Orthogonal stimuli are tested in every block) and across all 12 experiments (including Experiments 4 and 5 where Orthogonal stimuli were not presented in the first two testing blocks).
(XLSX)
